# Complete Genome Sequence of *Staphylococcus epidermidis* ATCC 12228 Chromosome and Plasmids, Generated by Long-Read Sequencing

**DOI:** 10.1128/genomeA.00954-17

**Published:** 2017-09-07

**Authors:** Kyle S. MacLea, Ariel M. Trachtenberg

**Affiliations:** a Biology Program, University of New Hampshire, Manchester, New Hampshire, USA; b Biotechnology Program, University of New Hampshire, Manchester, New Hampshire, USA; c Department of Life Sciences, University of New Hampshire, Manchester, New Hampshire, USA

## Abstract

*Staphylococcus epidermidis* ATCC 12228 was sequenced using a long-read method to generate a complete genome sequence, including some plasmid sequences. Some differences from the previously generated short-read sequence of this nonpathogenic and non-biofilm-forming strain were noted. The assembly size was 2,570,371 bp with a total G+C% content of 32.08%.

## GENOME ANNOUNCEMENT

Among the Gram-positive staphylococci, *Staphylococcus aureus* is the most well-known pathogen, contributing to dangerous human and animal infections, including septicemia, as well as foodborne intoxication. Among other members of the genus, some strains of the common human skin bacterium *Staphylococcus epidermidis* are associated with serious nosocomial infections (1), and others, such as *S. epidermidis* ATCC 12228, are common commensals not associated with pathogenicity (2). Many genome sequences are available for *S. epidermidis*, with 389 previously reported in GenBank, of which 11 were complete genome sequences generated with short-read methods, such as the Illumina platform, or long-read sequencing methodologies, such as the PacBio platform (3–5). Here, we report the first sequence generated for *S. epidermidis* ATCC 12228 using long-read technology after its initial report using a short-read method in 2003 (2).

*S. epidermidis* 12228 was obtained from Thermo Fisher Scientific in lyophilized form and rehydrated, and a culture was grown from an isolated colony on a tryptic soy agar plate in tryptic soy broth at 30°C for 72 h. The Genomic-tip 500/G kit (Qiagen, Valencia, CA, USA) was used according to the manufacturer’s instructions to isolate genomic DNA (gDNA). Purified gDNA of *S. epidermidis* 12228 was sequenced at the Institute for Genome Studies, University of Maryland, on a single PacBio (Pacific Biosciences, Menlo Park, CA, USA) RS II P6-C4 single-molecule real-time (SMRT) cell using a PacBio long-insert library after size selection to capture both plasmid and main chromosome sequences. The sequencing run resulted in a total of 155,545 long reads with a mean length of 6,023 bp, which represented an approximately 25-fold sequence coverage after read assembly. The generated genome size was 2,570,371 bp split into 6 contigs: the main chromosome of 2,497,508 bp and plasmids of 37,770 bp (pAMT1), 23,530 bp (pAMT2), 7,554 bp (pAMT3), 2,390 bp (pAMT4), and 1,619 bp (pAMT5). The G+C content of 32.08% was very close to the 32.1% determined by Illumina sequencing of the *S. epidermidis* 12228 genome (2).

Assembly of the genome was undertaken using the Celera version 8.1 assembler. Annotation of the genome used the NCBI Prokaryotic Genome Annotation Pipeline process (6), identifying a total of 2,545 genes, 2,462 coding sequences, 83 RNA genes (7 copies of 5S rRNAs, 6 of 16S rRNAs, 6 of 23S rRNAs, 60 tRNAs, and 4 noncoding RNAs), and 85 pseudogenes. Although this new assembly did not capture as many plasmids (5 versus 6) and generated a slightly smaller main chromosome (2,497,508 versus 2,564,615 bp) than the original Illumina assembly of *S. epidermidis* ATCC 12228 (2), it did reveal more of each of the categories of genes described above. This new long-read complete sequence provides an additional high-quality closed genome sequence for *S. epidermidis* ATCC 12228 that should be useful for better understanding the ability of some strains of *S. epidermidis* to cause disease in humans and animals or to adapt as commensal organisms.

### Accession number(s).

This whole-genome project has been deposited at DDBJ/ENA/GenBank under the accession numbers CP022247 to CP022252.
