# Correction for MacLea and Trachtenberg, “Complete Genome Sequence of Staphylococcus epidermidis AMT Chromosome and Plasmids, Generated by Long-Read Sequencing”

**DOI:** 10.1128/MRA.01231-20

**Published:** 2020-11-12

**Authors:** Kyle S. MacLea, Ariel M. Trachtenberg

**Affiliations:** a Biology Program, University of New Hampshire, Manchester, New Hampshire, USA; b Biotechnology Program, University of New Hampshire, Manchester, New Hampshire, USA; c Department of Life Sciences, University of New Hampshire, Manchester, New Hampshire, USA

## AUTHOR CORRECTION

Volume 5, no. 36, e00954-17, 2017, https://doi.org/10.1128/genomeA.00954-17.

The title should read as given in this correction, and Staphylococcus epidermidis ATCC 12228 (and Staphylococcus epidermidis 12228) should be changed to Staphylococcus epidermidis AMT throughout. Dr. Jesper Larsen of the Reference Laboratory for Antimicrobial Resistance and Staphylococci at the Statens Serum Institut, Copenhagen, Denmark, performed multilocus sequence typing on the sequence deposited as part of the published announcement. Dr. Larsen correctly showed that the Staphylococcus epidermidis strain described as part of this work does not match known multilocus sequence type markers for S. epidermidis ATCC 12228. We have examined our stocks of the bacterium and believe that, while the correct ATCC 12228 strain was rehydrated and cultured, the material that was used for purification of genomic DNA was instead from another S. epidermidis strain from an environmental source. We apologize for the error and are submitting this correction to ensure reliable genomic data are available to the field. Simultaneous with this correction, all database entries will be changed to reflect this new information. We will designate this new strain S. epidermidis strain AMT.

